# Detection of statistically significant network changes in complex biological networks

**DOI:** 10.1186/s12918-017-0412-6

**Published:** 2017-03-04

**Authors:** Raghvendra Mall, Luigi Cerulo, Halima Bensmail, Antonio Iavarone, Michele Ceccarelli

**Affiliations:** 1 QCRI - Qatar Computing Research Institute, HBKU, Doha, Qatar; 2 Department of Science and Technology, University of Sannio, Benevento, Italy; 3 BioGeM, Institute of Genetic Research “Gaetano Salvatore”, Ariano Irpino (AV), Italy; 4 Department of Neurology, Department of Pathology, Institute for Cancer Genetics, Columbia University Medical Center, New York, USA

**Keywords:** Differential networks, Gene regulatory network inference, Master regulators

## Abstract

**Background:**

Biological networks contribute effectively to unveil the complex structure of molecular interactions and to discover driver genes especially in cancer context. It can happen that due to gene mutations, as for example when cancer progresses, the gene expression network undergoes some amount of localized re-wiring. The ability to detect statistical relevant changes in the interaction patterns induced by the progression of the disease can lead to the discovery of novel relevant signatures. Several procedures have been recently proposed to detect sub-network differences in pairwise labeled weighted networks.

**Methods:**

In this paper, we propose an improvement over the state-of-the-art based on the Generalized Hamming Distance adopted for evaluating the topological difference between two networks and estimating its statistical significance. The proposed procedure exploits a more effective model selection criteria to generate *p*-values for statistical significance and is more efficient in terms of computational time and prediction accuracy than literature methods. Moreover, the structure of the proposed algorithm allows for a faster parallelized implementation.

**Results:**

In the case of dense random geometric networks the proposed approach is 10-15x faster and achieves 5-10% higher AUC, Precision/Recall, and Kappa value than the state-of-the-art. We also report the application of the method to dissect the difference between the regulatory networks of IDH-mutant versus IDH-wild-type glioma cancer. In such a case our method is able to identify some recently reported master regulators as well as novel important candidates.

**Conclusions:**

We show that our network differencing procedure can effectively and efficiently detect statistical significant network re-wirings in different conditions. When applied to detect the main differences between the networks of IDH-mutant and IDH-wild-type glioma tumors, it correctly selects sub-networks centered on important key regulators of these two different subtypes. In addition, its application highlights several novel candidates that cannot be detected by standard single network-based approaches.

**Electronic supplementary material:**

The online version of this article (doi:10.1186/s12918-017-0412-6) contains supplementary material, which is available to authorized users.

## Background

The omni-presence of complex networks is reflected in wide variety of domains including social networks [1, 2], web graphs [3], road graphs [4], communication networks [5], financial networks [6] and biological networks [7–9]. Although we focus on biological networks many aspects of the method proposed in this paper can also be applied for networks in other contexts. In cancer research, the comparison between gene regulatory networks, protein interaction networks, and DNA methylation networks is performed to detect differences between two conditions, such as, healthy and disease [10, 11]. This can lead to discovery biological pathways related to the disease condition, and, in case of cancer, the gene regulatory changes as the disease progresses [12–14].

A central problem in cell biology is to model functional networks underlying interactions between molecular entities from high throughput data. One of the main question is how the cell globally changes its behavior in response to external stimuli or what is the effect of alterations such as, driver somatic mutations and changes in copy number. Signatures of differentially expressed and/or methylated genes are the downstream effect of global cell de-regulation in different conditions such as cancer subtypes. Therefore, it is argued that driver mutations activate functional pathways described by different global re-wiring of the underlying gene regulatory network.

The identification of significant changes induced by the presence or the progression of disease can help to discover novel molecular diagnostics and prognostic signatures. For example, it is known that, according to the mutation of the gene IDH [15, 16], the majority of malignant brain tumors can be divided two main macro-categories, which can be further divided in seven molecular and clinically distinct subtypes [17]. These two macro-groups are characterized by highly different global expression and epigenomic profiles. Hence, one of the main questions to understand the molecular basis of diseases is how to identify significant changes in the regulatory structure in different conditions.

Various techniques have been developed to compare two graphs including graph matching and graph similarity algorithms [18–20]. However, the problem addressed in this paper is different from popular graph theory problems including graph isomorphism [21] and sub-graph matching [22]. Here the goal is to identify statistically significant differences between two weighted networks (with or without labels).

One common statistic used to distinguish one graph, *A* from another *B*, having the same number of nodes *N*, is the Mean Absolute Difference (MAD) metric, defined as: $d(A,B)=\frac {1}{N(N-1)}\sum _{i\neq j}|a_{ij}-b_{ij}|$, where *a*_*ij*_ and *b*_*ij*_ are edge weights corresponding to the topology of networks *A* and *B*. This distance measure is equivalent to the Hamming distance [23] and has been extensively used in literature to compare networks [24, 25]. Another statistic used to test association between networks is the Quadratic Assignment Procedure (QAP) defined as: $Q(A,B) = \frac {1}{N(N-1)} \sum _{i=1} \sum _{j=1} a_{ij}b_{ij}$. The QAP metric is used in a permutation-based procedure to differentiate two networks [26, 27]. Ruan et al. showed that these metrics are not always sensitive to subtle topological variations [28].

Our aim is to detect statistically significant differences between two networks under the premise that any true topological difference between the two networks would involve only a small set of edges when compared to all the edges in the network. Recently, a Generalized Hamming Distance (GHD) based method was introduced to measure the distance between two labeled graphs [28], where it was shown that the GHD statistic is more robust than MAD and QAP metrics for identifying subtle variations in the topology of paired networks. In particular the authors showed that GHD permutation distribution follows a normal distribution with closed-form expression for first two moments under the null hypothesis that networks *A* and *B* are independent. Utilizing the moments, corresponding *p*-values were obtained in closed-form. They also propose a differential sub-network identification technique namely dGHD. The advantage of this technique is that – unlike previous differential network analysis techniques [25, 29, 30] – it provides a closed-form solution for *p*-values for the differential sub-network left after iterative removal of the least differential nodes. We propose an improvement over dGHD, namely Closed-Form approach that exploits the conditions for asymptotic normality which is computationally cheaper and attains better prediction performance than the dGHD algorithm. Computational efficiency and prediction accuracy is crucial in cancer contexts where networks have a large number of nodes and the topological difference is associated to few driver genes.

## Methods

### Preliminaries on generalized hamming distance

The Generalized Hamming Distance is a way to estimate the distance between two weighted graphs [28]. Let *A*=(*V*,*E*_*A*_) and *B*=(*V*,*E*_*B*_) be two graphs, with the same set of nodes *V*={1,…,*N*}, and different sets of edges, *E*_*A*_ and *E*_*B*_. The Generalized Hamming Distance (GHD) is defined as:

1 $$ \text{GHD}(A,B)=\frac{1}{\mathrm{N}(\mathrm{N}-1)}\sum\limits_{i,j,i\neq j} \left(a'_{ij}-b'_{ij}\right)^{2},  $$

where $a^{\prime }_{ij}$ and *b* *ij*′ are mean centered edge-weights defined as:

$$\begin{aligned} &a'_{ij}=a_{ij}-\frac{1}{N(N-1)}\sum\limits_{i,j,i\neq j} a_{ij},\\ &b'_{ij}=b_{ij}-\frac{1}{N(N-1)}\sum\limits_{i,j, i\neq j} b_{ij} \end{aligned} $$

The edge weights, *a*_*ij*_ and *b*_*ij*_, depend on the topology of the network and provide a measure of connectivity between every pair of nodes *i* and *j* in *A* and *B*. Different metrics have been adopted to measure the connectivity between pairs of nodes, including: topological overlap (TO) [31, 32], cosine similarity and Pearson correlation [33]. In our experiments, we used the cosine similarity to capture first order interactions between the nodes in the network. Cosine similarity computation scales well for large sparse networks and can be used in place of TO, as it has nearly perfect correlation with it.

Given two networks *A* and *B*, a permutation *π* of the labels of the vertices of *A* (keeping the edges unchanged) generates a permuted network *A*_*π*_. The quantity *G* *H* *D*_*π*_(*A*_*π*_,*B*) represents the test statistics of an inferential problem having as null hypothesis $\mathcal {H}_{o}$: *Graphs A and B are independent* [28]. The distribution of *G* *H* *D*_*π*_ can be obtained through an exhaustive calculation which can be approximated by a Monte Carlo approach. The authors of [28], indeed, simplified this calculation showing that under the null hypothesis it can be approximated well by a normal distribution with moments that can be obtained analytically.

This can be shown as:

2 $$ \frac{\text{GHD}(A_{\pi},B)-\mu_{\pi}}{\sigma_{\pi}} \sim \mathit{N}(0,1)  $$

where *μ*_*π*_ is the asymptotic value of the mean GHD statistic and *σ*_*π*_ is the asymptotic value of the standard deviation of GHD statistic computed between *A*_*π*_ and *B*. In order to calculate the *μ*_*π*_ and *σ*_*π*_ values we define:

$${}\begin{aligned} S_{a}^{t}=\sum\limits_{i=1}^{N} \sum\limits_{j=1,j\neq i}^{N} a_{ij}^{t}, t=1,2 \quad \text{and} \quad T_{a}=\sum\limits_{i=1}^{N} \left(\sum\limits_{j=1,j\neq i}^{N} a_{ij}\right)^{2} \\ S_{b}^{t}=\sum\limits_{i=1}^{N} \sum\limits_{j=1,j\neq i}^{N} b_{ij}^{t}, t=1,2 \quad \text{and} \quad T_{b}=\sum\limits_{i=1}^{N} \left(\sum\limits_{j=1,j\neq i}^{N} b_{ij}\right)^{2} \end{aligned} $$

Here $a_{ij}^{t}$ and $b_{ij}^{t}$ are the edge weights with the power *t*. Furthermore, we require the following terms:

$${}\begin{aligned} A_{a} = \left(S_{a}^{1}\right)^{2},\quad \! B_{a}=T_{a}\! - \!\left(S_{a}^{2}\right)\ \text{and}\ \ C_{a}=A_{a}\,+\,2\left(S_{a}^{2}\right)\,-\,4T_{a} \\ A_{b}=\left(S_{b}^{1}\right)^{2},\quad \! B_{b}=T_{b}\! -\!\left(S_{b}^{2}\right)\ \text{and}\ \ C_{b}=A_{b}\! +\! 2\left(S_{b}^{2}\right)\! -\! 4T_{b} \end{aligned} $$

Using these definitions the closed-form expression for mean *μ*_*π*_ and variance $\sigma _{\pi }^{2}$ are expressed as:

3 $$ \begin{aligned} \mu_{\pi}=&\frac{S_{a}^{2}+S_{b}^{2}}{N(N-1)}-\frac{2\left(S_{a}^{1}\right)\left(S_{b}^{1}\right)}{N^{2}(N-1)^{2}}, \\ \sigma_{\pi}^{2}=&\frac{4}{N^{3}(N-1)^{3}}\left[2\left(S_{a}^{2}\right)\left(S_{b}^{2}\right)+\frac{4(B_{a})(B_{b})}{N-2} \right.\\ &+\left.\frac{(C_{a})(C_{b})}{(N-2)(N-3)}-\frac{(A_{a})(A_{b})}{N(N-1)}\right] \end{aligned}  $$

Given a significance threshold *α* (e.g. 0.01), *p*-values >*α* indicate that there is no sufficient evidence to reject the null hypothesis ($\mathcal {H}_{o}$) that graphs *A* and *B* are independent. Hence, higher *p*-values indicate more probability that the two graphs under consideration are independent.

### Differential sub-network detection with GHD

The GHD distance is able to tell us to what extent are two graphs different but is not able to identify which parts of the graph are similar and which are different. In this work, we are interested in detecting which part of the graphs contribute to make the two graphs different. We call such different sub-graphs *differential sub-networks*.

The notion of differential sub-networks is based on the idea that when comparing two networks only a subset of edges would present altered interaction. The goal is to identify the set of nodes, namely *V* ^∗^, associated with such a subset of edges and the *p*-values *p* ^∗^ corresponding to the nodes in *V* ^∗^. This goal, formulated as a statistical test, requires that for such a subset *V* ^∗^ there is no sufficient evidence to reject the null hypothesis that the corresponding sub-networks $A^{*}(V^{*},E_{A^{*}})\phantom {\dot {i}\!}$ and $B^{*}(V^{*},E_{B^{*}})\phantom {\dot {i}\!}$ are statistically independent.

The idea here is to adopt an iterative technique to identify the set of nodes *V* ^∗^ which contributes more to the difference. We start from the dGHD algorithm proposed in [28]. The algorithm measures the edge connectivity with topological overlap metric and benefits from the closed-form solution of *p*-value (Eq. (3)). In the dGHD algorithm, an iterative procedure is followed where at each iteration the change in centralized GHD (cGHD) i.e. cGHD=GHD(*A*,*B*)−*μ*_*π*_ is estimated after the removal of one node. The node where the change in cGHD (i.e. difference in cGHD before and after removal of a node) is maximum is removed. The GHD statistic is computed for remaining sub-networks and the *p*-value is estimated. This process is repeated till a user specified minimal set size is reached or it is no-longer possible to have closed-form representation for *p*-values which happens for *N*≤3 as shown in Eq. 3. The *p*-values are then adjusted for multiple testing by controlling the false discovery rate [34].

The dGHD algorithm suffers from the following limitations: a) During the *i* ^*th*^ iteration, the GHD measure is calculated *N*−*i* times on different sub-graphs with an overall time complexity ∼*O*(*N* ^2^×|*E*|) where *E*=*E*_*A*_∪*E*_*B*_; b) The algorithm is prone to discovery more false positives since it uses the change in cGHD (*Δ*cGHD) as a model selection criterion. We overcome such limitations by proposing the following improvements:

1. *Remove nodes by exploiting the Closed-Form*. We use the idea that nodes which have similar topology in networks *A* and *B* will contribute the least to cGHD. So, we first calculate the closed-form contribution of each node in cGHD once using Eq. 4 and then iteratively remove nodes with least contributions. However, this process is continued till we observe that the *p*-value of the remaining sub-network becomes greater than a threshold *θ*.
2. *Using a different model selection criterion*. Once the *p*-value reaches *θ*, we follow a procedure similar to the dGHD algorithm but use the more intuitive criterion of selecting the node that when removed makes the cGHD value maximum rather than using the change in the cGHD value (before and after removal of a node) as a model selection criterion. By using this model selection criterion, we iteratively identify and remove that node whose contribution is least in the cGHD.
  The advantage of the Closed-Form approach is that we significantly reduce the computational complexity and improve the predictive performance. A simple alternative to the Closed-Form approach would be to sort all the nodes based on their contribution to cGHD and thus rank all the nodes based on their capability to differentiate the two networks with complexity (*O*(*N* log*N*)). However, then we will not be able to identify statistically different sub-networks between the two graphs as indicated in [28].

#### Closed-form approach

We propose a fast approach to perform differential sub-network analysis taking into consideration the contribution of each node to GHD and *μ*_*π*_. Using Eqs. (1) and (3) this can mathematically be represented as:

4 $$ {}{{\begin{aligned} \text{GHD}(A,B)(i)=&\frac{1}{N(N-1)}\!\left(\sum\limits_{j=1,j\neq i}^{N}(a'_{ij})^{2} \! +\!\! \sum\limits_{j=1,j\neq i}^{N} (b'_{ij})^{2}\! -\! \! \sum\limits_{j=1,j\neq i}^{N}\left(2a'_{ij}\!\times\! b'_{ij}\right)\right) \\ \mu_{\pi}(i)=&\frac{\left(\sum_{j=1,j\neq i}^{N}(a_{ij})^{2}+\sum_{j=1,j\neq i}^{N} (b_{ij})^{2}\right)}{N(N-1)}\! -\!\frac{2\left(\sum_{j=1,j\neq i}^{N} a_{ij}\right)\left(S_{b}^{1}\right)}{N^{2}(N-1)^{2}} \\ &-\!\frac{2\left(\sum_{j=1,j\neq i}^{N} b_{ij}\right)\left(S_{a}^{1}\right)}{N^{2}(N-1)^{2}}\! +\!\frac{2\!\left(\sum_{j=1,j\neq i}^{N} a_{ij}\right)\!\left(\sum_{k=1,k\neq i}^{N} b_{ik}\right)}{N^{2}(N-1)^{2}} \end{aligned}}}  $$

We observe that if we sum GHD(*A*,*B*)(*i*) and *μ*_*π*_(*i*)∀*i*∈*V*, we obtain GHD(*A*,*B*) and *μ*_*π*_. We use the idea that nodes which have similar topology in networks *A* and *B* will contribute the least to centralized GHD, i.e. GHD(*A*,*B*)−*μ*_*π*_. We calculate the Closed-Form contribution of each node in the centralized GHD (cGHD) once using Eq. (4) and then iteratively remove nodes with least contribution to the cGHD, i.e. nodes having similar topology in graphs *A* and *B*. Thus, we calculate cGHD once and sort all the nodes based on their contribution to the cGHD metric.

This process is continued till we observe that the *p*-value of the remaining sub-network becomes greater than a threshold *θ*. Once the *p*-value reaches *θ*, we estimate $\Delta _{V_{K}}=\text {GHD}\left (A(V_{K},E_{A}),B(V_{K},E_{B})\right)-\mu _{V_{K}}$ where $\mu _{V_{K}}$ is the mean of the permutation distribution for the nodes (*V*_*K*_) of the remaining sub-network. Furthermore, we define $\Delta _{V_{K|i}}$ as the value of cGHD after removal of node *i*. We adopt a different model selection criterion than that proposed in [28] to remove non-differential nodes. We use the intuitive criterion of selecting that node after removal of which the cGHD value becomes maximum, i.e. the node which was most similar in terms of topology for the paired-graphs. Finally, the obtained *p*-values are adjusted for multiple testing by controlling the false discovery rate [34]. Provided the paired-graphs *A* and *B*, the calculation of $\Delta _{V_{K|i}}$ can be done independently for each *i*. Details of the Closed-Form method is provided in Algorithm 1. The sensitivity of the Closed-Form approach with the parameter *θ* is demonstrated in Experimental Results section. Table 1 summarizes the improvements with respect to the dGHD algorithm in terms of time complexity.

**Table 1:** Time complexity comparison

dGHD	Closed-form
*O*(*N* ^2^|*E*|)	*O*(*N*|*E*|+*N* log(*N*)+*K* ^2^|*E*|)

Here *K* represents the number of nodes for which *p*-value is greater than *θ* and generally *K*≪*N*. An important remark is that the cGHD calculation after removal of each node can be done independently in parallel. So, in case we have *T* processors, the complexity of the proposed approach will reduce ≈ linearly w.r.t. *T*



#### Alternative procedure (fast approximation)

We propose an alternative procedure to the Closed-Form approach namely the Fast Approximation method where we first calculate the cGHD value without including the *i* ^*th*^ node, ∀*i*∈*V* once. This helps to estimate the cGHD value after removal of the *i* ^*th*^ node and can be performed in parallel. Our aim is to quickly discard those nodes after removal of which the cGHD value becomes large thereby removing nodes which were contributing least to the cGHD value. This helps to reduce the dependence between the two sub-networks by removing nodes which have similar topology in graphs *A* and *B*. Again, the idea is motivated by the premise that only a subset of nodes will form the differential sub-networks in graph *A* and *B*.

In this approach, we iteratively discard those nodes after removal of which the cGHD value becomes maximal till the *p*-value for the remaining sub-network reaches a threshold *θ*. Once the *p*-value reaches *θ*, we return back to the procedure of estimating $\Delta _{V_{K|i}} \forall i \in V_{K}$ as described in the Closed-Form approach. We use the same model selection criterion of selecting that node after removal of which the cGHD value becomes maximum as used in the Closed-Form approach. We then adjust the obtained *p*-values for multiple testing by controlling the false discovery rate [34]. We refer to this technique as a Fast Approximation to the dGHD [28]. We explain the Fast Approximation technique in detail in Algorithm 2.



From our experiments, we observe that the results of the Closed-Form approach and the Fast Approximation technique are identical. Although, in the case of Closed-Form approach, we calculate closed-form contribution of each node in the cGHD value and remove the node with least contribution, while in case of Fast Approximation we select that node after removal of which cGHD value becomes maximum, the ordered list $\mathcal {O}$ obtained for both the methods is identical. Moreover, the computational complexity of the Fast-Approximation technique is the same as that of Closed-Form approach.

### Inference of the glioma networks and master regulator analysis

We used the TCGA pan-glioma samples dataset including 1250 samples (463 IDH-mutant and 653 IDH-wild-type), 583 of which profiled with Agilent microarray and 667 with RNA-Seq Illumina HiSeq (REF) downloaded from the TCGA portal. The batch effects between the two platform were corrected using the COMBAT algorithm [35]. The final gene expression data matrix includes 12,985 genes and 1250 samples. We re-constructed two gene regulatory networks belonging to two different glioma subtypes: IDH-mutant and IDH-wild-type. Both networks were re-constructed with a four step procedure that follows ARACNe [36]: i) Computation of mutual information between gene expression profiles to determine interaction between Transcription Factors (TFs) and target genes [37]; ii) data processing inequality to filter out indirect relationships [36], iii) permutation test with 1000 re-samplings to keep only statistically significant relationships. We also assembled a global glioma network using all the available 1250 transcriptional profiles using the aforementioned method. In this last case we also used intersection with transcription factor (TF) binding sites to keep only relationships due to promoter binding. We used a set of 457 TF binding sites available in the MotifDB Bioconductor package.

Master Regulator Analysis (MRA) algorithm [38] was applied to the global glioma network in order to compute the statistical significance of the overlap between the regulon of each TF (i.e. its ARACNe inferred targets) and the differentially expressed gene list (Wilcoxon-Mann-Whitney test FDR≤0.05) between IDH-mutant and IDH-wild-type samples. Given a gene interaction network, generated by ARACNe and a gene phenotype signature (e.g. a set of differentially expressed genes), the MRA algorithm computes for each TF the enrichment of the phenotype signature in the regulon of that TF. The regulon of a TF is defined as its neighborhood in the gene interaction network. There are two different methods to evaluate the enrichment of the signature in the regulon. One method uses the statistical Fisher’s exact test, while the other approach uses Gene Set Enrichment Analysis (GSEA). Here we used this last method.

A Master Regulator (MR) gene is a TF which regulon exhibit a statistical significant enrichment of the given phenotype signature.

### Validation in the Rembrandt dataset

We used an independent dataset to perform the same analysis of network differencing between IDH-mutant and IDH-wild-type gliomas and check the the overlap between the two analyses. Raw gene expression (Affymetrix U133 Plus 2.0) from the publically available Repository for Molecular Brain Neoplasia Data (Rembrandt) (https://caintegrator.nci.nih.gov/rembrandt/) included 444 samples divided in 218 Glioblastoma, 148 Astrocytoma, 67 Oligodendrogliomas and 11 mixed histologies. Expression subtype and IDH status was inferred from gene expression following the procedure in [39] resulting in 153 wild-type and 162 mutant samples. These two set of expression profiles were used to generate two regulatory networks using the same approach reported above.

## Results and discussion

For all our experiments, we used the Closed-Form approach (since results obtained from Closed-Form and Fast-Approximation techniques are identical) and compare it with the dGHD method [28].

### Cosine similarity and topological overlap

The one-step topological overlap measure used to estimate the edge weights is defined as:

5 $$ a_{ij}=\frac{\sum_{l\neq i,j}A_{il}A_{lj}+A_{ij}}{\text{min}\left(\sum_{l\neq i}A_{il}-A_{ij},\sum_{l\neq j} A_{lj}-A_{ij}\right) +1}  $$

In this work we use the cosine similarity to calculate the edge weights *a*_*ij*_. The cosine similarity takes into consideration one-step neighborhood of nodes *i* and *j* while constructing the edge weight and is very efficient to calculate for sparse matrices. The weights *a*_*ij*_ are estimated as follows:

6 $$ a_{ij}=\frac{\sum_{l}A_{il}A_{jl}}{\sqrt{\sum_{l}A_{il}^{2}}\sqrt{\sum_{l}A_{jl}^{2}}}  $$

where *A*_*ij*_ represents the adjacency matrix.

We perform an experiment to calculate the correlation between the one-step topological measure and the cosine similarity measure. For this experiment, we generated 250 random geometric networks using *N*=250 and the connectivity parameter *d*=0.15.

**Fig. 1:**
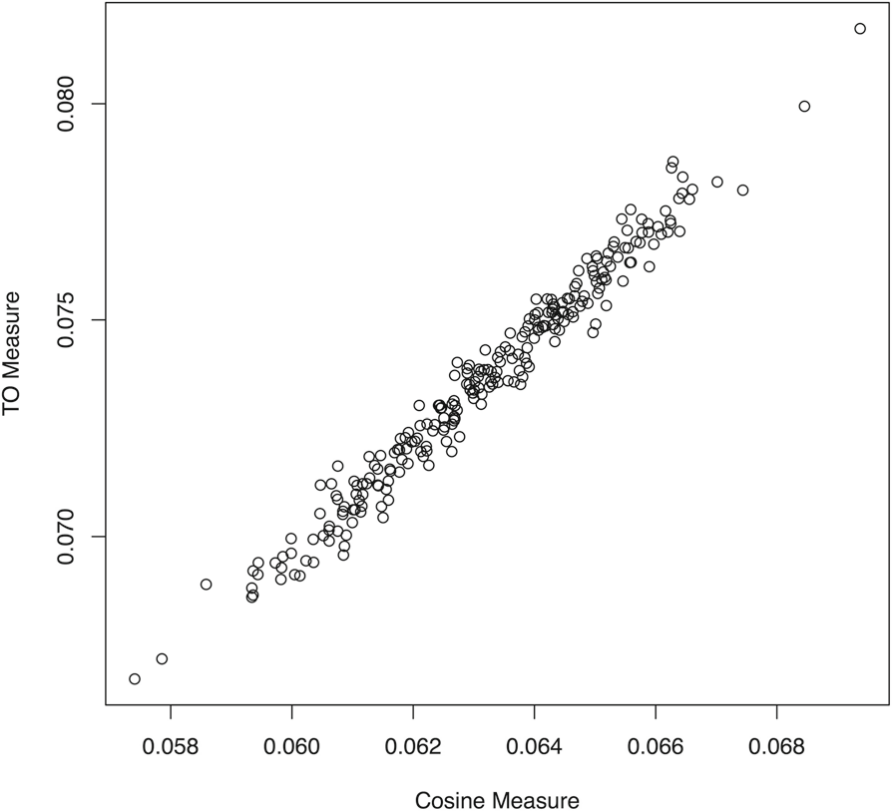
Correlation between topological overlap and cosine similarity on 250 random networks

Figure 1 shows that the cosine similarity metric is nearly perfectly correlated (Pearson correlation = 0.952) to the topological overlap measure.

### Sensitivity to *θ*

In this experiment, we check the sensitivity of the proposed Closed-Form approach w.r.t. the heuristic *θ*. For this experiment, we first generated 100 random geometric (RG) networks. In a RG network nodes are generated by uniformly sampling *N* points on [ 0,1]^2^. An edge is then drawn between these points if the Euclidean distance between the points is less than a parameter *d*. This parameter *d* controls the density of the RG network where smaller values of *d* result in sparse networks while larger values of *d* generates dense networks. In our case, we conducted experiments using two different settings. In the first case, we use *d*=0.15, while in the second setting, we use *d*=0.3. For both experiments we fix *N*=250. For each value of *d* and for each generated RG network *A*, we permute the first 50 rows and columns of the network to generate network *B*. Therefore, the first 50 nodes in networks *A* and *B* form the gold-standard.

**Fig. 2:**
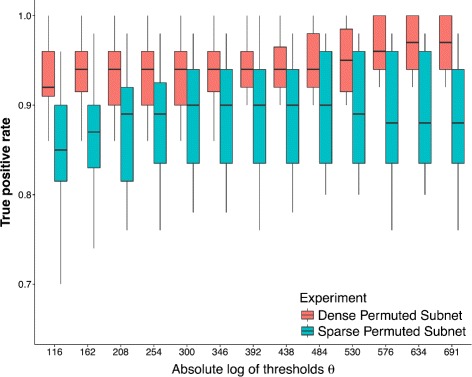
Sensitivity Analysis of Parameter *θ*. The boxplots represents the distribution of True Positive Rate (TPR) identified by Closed-Form approach for 100 random runs of the experiment

In order to test the sensitivity of the proposed approach w.r.t. *θ*, we estimate the fraction of permuted nodes correctly identified by the Closed-Form method for various values of *θ*. We used a grid of *θ* values varying from *Θ*={10^−50^,…,10^−300^} in multiplicative steps of 10^−20^. The goal of this experiment is to show that the fraction of correctly identified nodes w.r.t. various *θ*∈*Θ* remains nearly constant for smaller values of *θ*. Figure 2 shows the result for RG networks with density parameter *d*=0.15 and *d*=0.3. From Fig. 2, we observe that the median fraction of permuted nodes identified by the proposed approaches increases slowly before it converges to a nearly constant value as we decrease the threshold *θ* (i.e. increase absolute log of threshold *θ*).

From this experiment, we conclude that the fraction of truly differential nodes identified by the proposed methods increases as we decrease the threshold *θ* before it starts to converge for smaller values of threshold *θ*.

We performed further experiments using different *θ* for various values of *N* and observed that threshold *θ* behaves similarly independent of the value of *N*. We used the *θ*=10^−50^ as heuristic cut-off for future experiments.

### Predictive performance comparison

**Experimental Setup**: The next simulation study that we carried out was to compare the predictive performance of the proposed approach w.r.t. the dGHD [28] technique. For this experiment, we generate 100 RG networks with *N*=1,000. For the first experiment we fix the density parameter *d*=0.15 and permute first 100 nodes in network *A* to obtain network *B*. Thus, these first 100 nodes form the differential sub-network for the paired networks *A* and *B*.

In the second case, we use the density parameter *d*=0.3 to generate the edges for network *A*. We then generate a small RG network with 100 nodes using density parameter *d* ^′^=0.5. This small dense sub-network is then used to replace the network formed by first 100 nodes in the original network *A* to form network *B*. Thus, in the second experiment, these 100 nodes form the differential sub-network for the paired networks *A* and *B*. This kind of mechanism can appear in real-life networks, for example, in case of cancer the transcription activity of some set of genes might get enhanced or suppressed in patients resulting in more or fewer edges in a sub-network of the gene or DNA methylation network. Hence, the networks generated in the first case are much sparser in comparison to the networks generated in the second case.

**Evaluation Metrics**: We define the following terms to be used in our analysis:

- True Positives (TP) - Refers to the nodes that are correctly identified as part of a differential network.
- False Positives (FP) - Refers to the nodes that are incorrectly identified as part of a differential network.
- False Negatives (FN) - Refers to the nodes that are part of the differential sub-network but are not identified correctly as part of the sub-network.
- True Negatives (TN) - Refers to the nodes that are correctly identified as nodes which are not part of the differential sub-network *A* ^∗^ and *B* ^∗^.

**Fig. 3:**
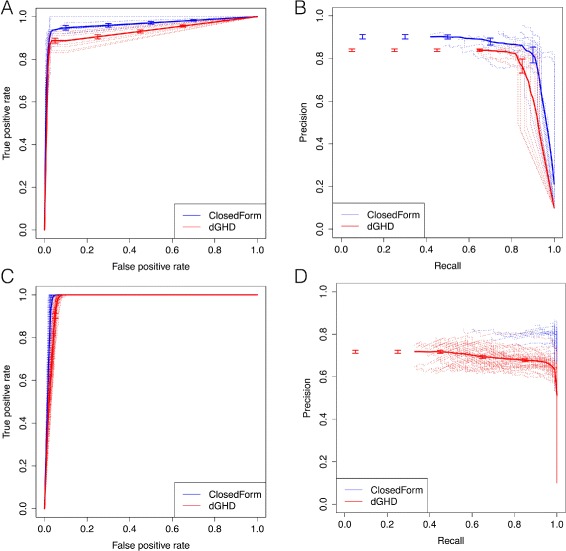
Comparison of proposed Closed-Form approach with dGHD algorithm. Figure **a** and **b** correspond to the ROC and PR plot for permuted sub-network (*d*=0.15) respectively. Figure **c** and **d** represents the ROC and PR plot corresponding to denser sub-network (*d*=0.3 and *d* ^′^=0.5) respectively. Clearly, the Closed-Form technique has better performance than the dGHD algorithm

**ROC and PR curve comparisons**: We generate two set of plots including the receiver operating characteristic (ROC) curves and the precision-recall (PR) curves. To generate the plots as shown in Fig. 3, we use the ‘ROCR’ [40] package in R. It generates relatively smooth curves by automatically using different thresholds to estimate the true positive rate i.e. $\frac {n(TP)}{n(TP)+n(FN)}$ and the false positive rate i.e. $\frac {n(FP)}{n(FP)+n(TN)}$ for ROC plot and precision i.e. $\frac {n(TP)}{n(TP)+n(FP)}$ and recall i.e. $\frac {n(TP)}{n(TP)+n(FN)}$ for the PR plot. Here we use the true positive rate (TPR) and Recall interchangeably. Here *n*(·) represents the total number of nodes. For generating the plots we used the adjusted *p*-value lists as obtained from the Closed-Form and dGHD approaches without specifying any threshold to generate smooth curves.

The data in Fig. 3 a and c shows that Closed-Form approach achieves better performance in case of differential sub-networks formed by permuted nodes and sub-networks with higher density. One of the reasons for relatively poor performance of the dGHD approach is that it has low true positive rate (TPR) and a high false positive rate (FPR) when the network has more edges. This is also reflected by the relatively low Recall and Precision values for the dGHD algorithm in Table 2 when *d*=0.3 and *d* ^′^=0.5. From Fig. 3 c, we can observe that the performance of both the dGHD and Closed-Form algorithm improves w.r.t. ROC when the differential sub-network is denser than the remaining network. However, the gap between the PR curves of Closed-Form and dGHD methods increases when the differential sub-network is denser.

**Table 2:** Comparison of proposed Closed-Form (CF) approach with dGHD algorithm. We compared the proposed Closed-Form approach with dGHD, Louvain, Infomap and Spinglass techniques w.r.t. various evaluation metrics for random geometric (RG) and power law (PL) networks

Parameters	Method	AUC_ROC	Precision	Recall	Accuracy	Specificity	Kappa	Time
		Mean ± Sd	Mean ± Sd	Mean ± Sd	Mean ± Sd	Mean ± Sd	Mean ± Sd	Mean
*d*=0.15 (RG)	**CF**	0.935 ± 0.051	**0.849 ± 0.037**	0.846 ± 0.102	**0.969 ± 0.011**	**0.983 ± 0.004**	0.828 ± 0.068	0.078
*d*=0.15 (RG)	**dGHD**	0.926 ± 0.018	0.793 ± 0.021	0.878 ± 0.036	0.965 ± 0.005	0.974 ± 0.003	0.813 ± 0.026	1.0
*d*=0.15 (RG)	**Louvain**	**0.980 ± 0.016**	0.767 ± 0.052	**1.0 ± 0.0**	0.965 ± 0.028	0.960 ± 0.031	**0.841 ± 0.113**	**0.012**
*d*=0.15 (RG)	**Infomap**	0.843 ± 0.012	0.262 ± 0.015	**1.0 ± 0.0**	0.718 ± 0.022	0.685 ± 0.024	0.304 ± 0.024	0.018
*d*=0.15 (RG)	**Spinglass**	0.832 ± 0.011	0.249 ± 0.012	**1.0 ± 0.0**	0.699 ± 0.018	0.665 ± 0.021	0.285 ± 0.020	0.85
*d*=0.15, *d* ^′^=0.3	**CF**	0.927 ± 0.048	0.839 ± 0.031	0.862 ± 0.098	0.969 ± 0.008	**0.982 ± 0.005**	0.825 ± 0.054	0.081
*d*=0.15, *d* ^′^=0.3	**dGHD**	0.922 ± 0.022	0.806 ± 0.027	0.868 ± 0.045	0.966 ± 0.006	0.977 ± 0.004	0.816 ± 0.032	1.0
*d*=0.15, *d* ^′^=0.3	**Louvain**	**0.978 ± 0.018**	**0.887 ± 0.137**	0.974 ± 0.042	**0.982 ± 0.018**	**0.982 ± 0.023**	**0.916 ± 0.083**	**0.013**
*d*=0.15, *d* ^′^=0.3	**Infomap**	0.849 ± 0.008	0.269 ± 0.009	**1.0 ± 0.0**	0.728 ± 0.015	0.698 ± 0.016	0.316 ± 0.016	0.020
*d*=0.15, *d* ^′^=0.3	**Spinglass**	0.859 ± 0.009	0.284 ± 0.013	**1.0 ± 0.0**	0.747 ± 0.016	0.719 ± 0.017	0.339 ± 0.019	0.92
*d*=0.3 (RG)	**CF**	**0.877 ± 0.067**	**0.714 ± 0.075**	0.789 ± 0.135	**0.947 ± 0.016**	**0.975 ± 0.011**	**0.716 ± 0.099**	0.083
*d*=0.3 (RG)	**dGHD**	0.724 ± 0.029	0.645 ± 0.049	0.577 ± 0.059	0.921 ± 0.007	0.971 ± 0.006	0.504 ± 0.051	1.0
*d*=0.3 (RG)	**Louvain**	0.866 ± 0.019	0.406 ± 0.061	**1.0 ± 0.0**	0.850 ± 0.034	0.833 ± 0.038	0.505 ± 0.072	**0.013**
*d*=0.3 (RG)	**Infomap**	0.677 ± 0.011	0.147 ± 0.004	**1.0 ± 0.0**	0.419 ± 0.019	0.354 ± 0.022	0.100 ± 0.008	0.021
*d*=0.3 (RG)	**Spinglass**	0.678 ± 0.011	0.148 ± 0.004	**1.0 ± 0.0**	0.420 ± 0.018	0.355 ± 0.021	0.100 ± 0.008	0.90
*d*=0.3, *d* ^′^=0.5	**CF**	**0.979 ± 0.005**	**0.771 ± 0.061**	0.930 ± 0.082	**0.965 ± 0.012**	**0.969 ± 0.011**	**0.821 ± 0.062**	0.09
*d*=0.3, *d* ^′^=0.5	**dGHD**	0.848 ± 0.071	0.700 ± 0.038	0.731 ± 0.148	0.941 ± 0.010	0.964 ± 0.009	0.672 ± 0.078	1.0
*d*=0.3, *d* ^′^=0.5	**Louvain**	0.932 ± 0.029	0.478 ± 0.118	**1.0 ± 0.0**	0.879 ± 0.054	0.866 ± 0.059	0.582 ± 0.128	**0.014**
*d*=0.3, *d* ^′^=0.5	**Infomap**	0.674 ± 0.010	0.145 ± 0.004	**1.0 ± 0.0**	0.413 ± 0.018	0.348 ± 0.020	0.097 ± 0.008	0.023
*d*=0.3, *d* ^′^=0.5	**Spinglass**	0.711 ± 0.007	0.162 ± 0.003	**1.0 ± 0.0**	0.481 ± 0.013	0.423 ± 0.014	0.128 ± 0.006	0.94
*α*=2 (PL)	**CF**	**0.797 ± 0.046**	**0.307 ± 0.307**	0.792 ± 0.099	**0.801 ± 0.018**	**0.349 ± 0.051**	**0.802 ± 0.022**	0.09
*α*=2 (PL)	**dGHD**	**0.797 ± 0.013**	0.294 ± 0.009	0.809 ± 0.027	0.787 ± 0.008	0.333 ± 0.015	0.784 ± 0.009	1.0
*α*=2 (PL)	**Louvain**	0.780 ± 0.014	0.212 ± 0.010	**1.0 ± 0.0**	0.703 ± 0.018	0.272 ± 0.016	0.690 ± 0.011	**0.015**
*α*=2 (PL)	**Infomap**	0.665 ± 0.013	0.141 ± 0.004	**1.0 ± 0.0**	0.603 ± 0.018	0.162 ± 0.012	0.484 ± 0.019	0.026
*α*=2 (PL)	**Spinglass**	0.687 ± 0.014	0.153 ± 0.006	**1.0 ± 0.0**	0.645 ± 0.021	0.194 ± 0.011	0.527 ± 0.016	0.90
*α*=3 (PL)	**CF**	**0.825 ± 0.019**	**0.345 ± 0.015**	0.825 ± 0.035	**0.826 ± 0.007**	**0.402 ± 0.024**	**0.826 ± 0.004**	0.085
*α*=3 (PL)	**dGHD**	0.808 ± 0.027	0.327 ± 0.018	0.799 ± 0.050	0.816 ± 0.008	0.375 ± 0.031	0.817 ± 0.004	1.0
*α*=3 (PL)	**Louvain**	0.774 ± 0.015	0.233 ± 0.011	**1.0 ± 0.0**	0.736 ± 0.019	0.301 ± 0.009	0.732 ± 0.019	**0.015**
*α*=3 (PL)	**Infomap**	0.670 ± 0.014	0.168 ± 0.005	**1.0 ± 0.0**	0.635 ± 0.017	0.210 ± 0.014	0.532 ± 0.014	0.027
*α*=3 (PL)	**Spinglass**	0.694 ± 0.013	0.179 ± 0.007	**1.0 ± 0.0**	0.670 ± 0.023	0.232 ± 0.012	0.571 ± 0.017	0.94

Bold represents the best results

**Fig. 4:**
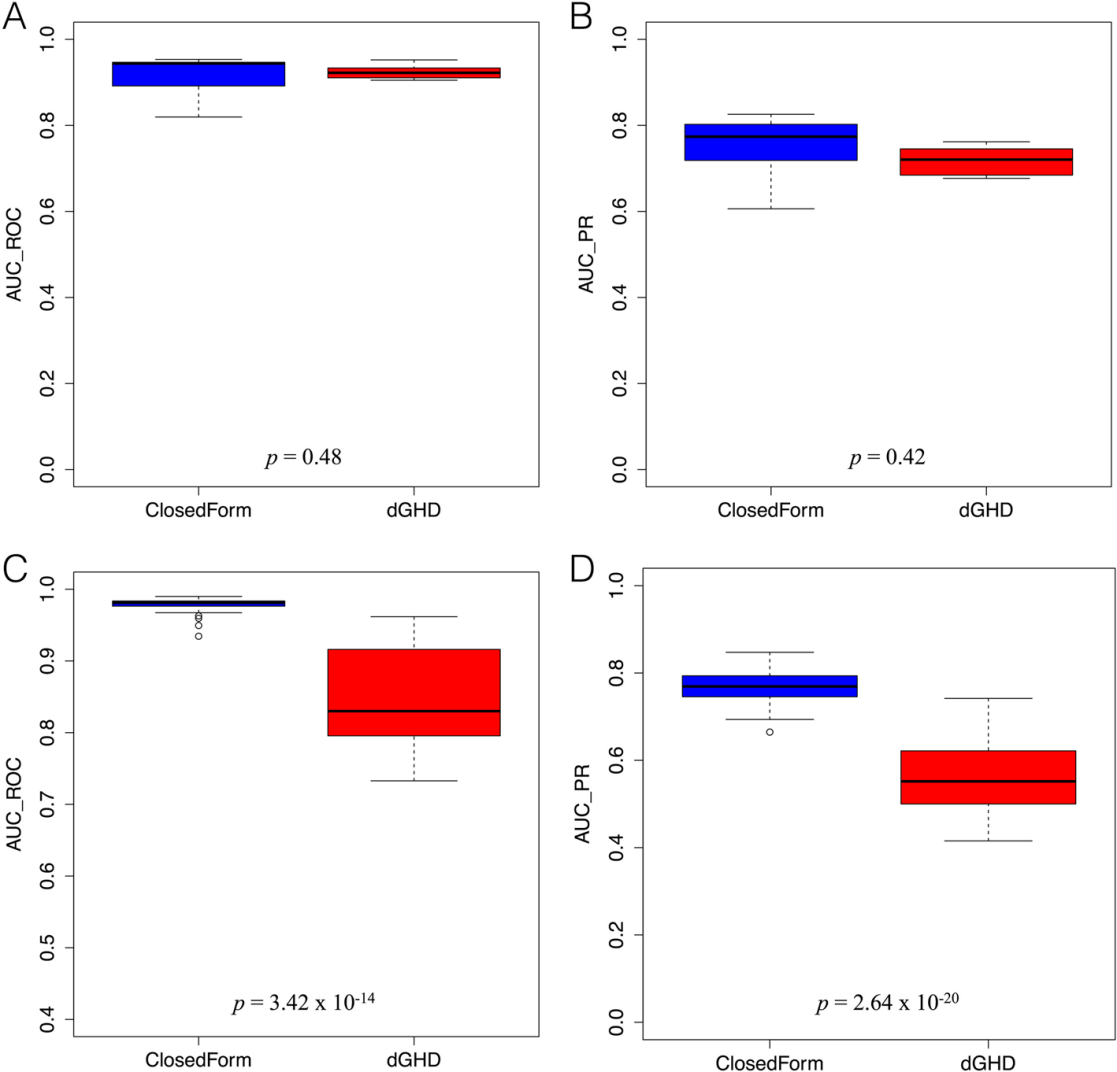
Comparison of proposed Closed-Form approach with dGHD method w.r.t. AUC _*ROC*_ and AUC _*PR*_ for 100 random runs of the experiment. These metrics are calculated using *p*-value 0.01 as cut-off. Figure **a** and **b** correspond to the AUC _*ROC*_ and AUC _*PR*_ for permuted sub-network (*d*=0.15) respectively. Figure **c** and **d** represents the AUC _*ROC*_ and AUC _*PR*_ corresponding to denser sub-network (*d*=0.3 and *d* ^′^=0.5) respectively

**AUC comparison**: For all further simulated experiments, we use *p*-value 0.01 as cut-off in order to determine TP, TN, FP and FN respectively. We also evaluated the area under the ROC curve (AUC_ROC [41]) and area under PR curve (AUC_PR [41]) for 100 runs of Closed-Form and dGHD methods (using *p*-value 0.01 as cut-off) as shown in Fig. 4.

We observe from Fig. 4 a and b that the dGHD method has lower variance w.r.t. AUC_ROC and AUC_PR metrics in comparison to Closed-Form approach in the case of permuted differential sub-network. However, in case of denser differential sub-network, the Closed-Form approach has much smaller variance in comparison to dGHD algorithm w.r.t. AUC_ROC and AUC_PR metrics as depicted in Fig. 4 c and d respectively. This suggests that the performance of Closed-Form technique is better than dGHD method when differential sub-networks are formed either using permuted nodes or higher density. In order to test for significance we performed the Student’s t-test under the null that the difference in the mean values of the two ROC distributions is zero i.e. $\mu _{AUC\_ROC_{A}}-\mu _{AUC\_ROC_{B}}=0$. At a significance level of 5%, we obtain *p*-value of 0.48 in case of permuted sub-network, thereby accepting the null i.e. the difference between the two distributions is not significant. In the case of paired networks with a denser differential sub-network (i.e. *d* ^′^=0.5), we obtain *p*-value of 3.42×10^−14^ for the Student’s t-test, thereby rejecting the null. Similarly for the two PR distributions we obtained *p*-value of 0.42 in case of permuted sub-network and *p*-value of 2.64×10^−20^ for the denser differential sub-network.

### Comparison with community detection techniques

The task of identifying differential sub-networks can also be rephrased as one of finding heavy sub-networks on a single network (say C) constructed by considering the absolute difference in the edge weights between the topological graph of network A and the topological graph of network B i.e. *C*_*ij*_=∥*a*_*ij*_−*b*_*ij*_∥,∀*i*,*j*∈*V*. This problem can then be construed as one of identifying dense modules in the network C i.e. from the previous experiments we want to discover a module corresponding to the set of nodes which have permuted or identify the denser sub-network forming the differential sub-network as a module.

The task of identifying dense/heavy modules in a network (C) is often referred as community detection or graph partitioning or graph clustering. There is a plethora of research associated with the problem of community detection including [42–49]. Several of these methods such as jActiveModules [50] and Spinglass algorithm [45] have also been applied to identify biologically meaningful modules (like functional modules, protein complexes, disease associated genes etc.) in biological networks as shown in [51, 52]. For our task of identifying dense modules in network C we applied 3 different community detection methods namely Louvain [43], Infomap [44] and Spinglass [45] techniques to have a comprehensive comparison with the proposed Closed-Form approach. We used the implementation of these methods available in the ‘igraph’ package in R and run each of these methods at their default settings.

We used the same set of RG networks as in the previous experiments to have a comparison with the community detection techniques. Since we are considering the difference in the topology of networks A and B in network C, we remove all the similarity between the two networks and the module with the maximum internal volume (i.e. total weight of edges within the community) is the one capturing the maximum difference between the topologies of networks A and B. Hence, we consider the densest inferred module as the one comprising the differential sub-network and label all the nodes belonging to this cluster as differential while all the other modules are considered non-differential. Using this notion to label the inferred communities, we compare the results obtained for the 3 different community detection techniques w.r.t. the gold standard (i.e. the actual set of labeled nodes which either belong to the permuted sub-network or belong to the denser sub-network) in a binary classification framework [53, 54]. These results are integrated in Table 2 along with the results of dGHD technique and the proposed Closed-Form (CF) approach. We assess the results obtained from the 3 community detection methods w.r.t. several quality metrics commonly used for binary classification including Precision, Recall, Kappa, Accuracy, Specificity, AUC_*ROC* and computational time. From Table 2, we observe that the Louvain method clearly outperforms the Infomap and Spinglass techniques in correctly identifying the differential sub-network as a module with respect to the various evaluation metrics.

### Simulated result analysis

Finally, the summary Table 2 highlights the computational efficiency and better predictive capabilities of the proposed technique in comparison to dGHD algorithm. For this comparison, we report the results obtained on 100 random runs of RG networks with *N*=1000,*d*=0.15 and *d*=0.3 respectively, where the first 100 nodes are permuted. We also report results when the first 100 nodes form the denser differential sub-networks i.e. in experiments where *d*=0.15 use *d* ^′^=0.3 to form denser sub-network and where *d*=0.3 use *d* ^′^=0.5 to form denser sub-network. We also conducted experiments on undirected Power Law (PL) graphs using *N*=1000 and *E*=10,000 with power law exponents *α*={2,3} respectively. We permuted the first 100 nodes of each PL network (*B*) to form the permuted network (*A*). We performed 100 random runs and report the mean values for various evaluation metrics.

Table 2 compares the Closed-Form, Louvain, Infomap, Spin-glass and dGHD techniques w.r.t. various standard evaluation metrics like AUC, Precision, Recall, Accuracy, Specificity, Kappa statistic and computational Time for all the simulation experiments. Higher values of these evaluation metrics represents better quality results. Here the time required by dGHD algorithm is normalized to 1 and the time required by the other algorithms is scaled by the same normalization factor.

We observe from Table 2 that the Closed-Form approach performs exceedingly well in case of experiments on denser RG networks (*d*=0.3) and PL graphs. It emerges as the best method on these networks for various evaluation metrics. For this configuration, in case of both permuted and denser differential sub-networks, the mean AUC_ROC of Closed-Form approach is at least 10% higher than the dGHD algorithm. This is also reflected in higher values of Precision (0.714 and 0.771) and Recall (0.789 and 0.930) metrics for Closed-Form approach in comparison to low values of Precision (0.645 and 0.7) and Recall (0.577 and 0.731) for the dGHD algorithm in case of these experiments.

However, in case of sparse networks where its relatively easier to identify differential sub-networks ([28]), both Closed-Form and dGHD method have similar predictive performance. For sparse networks, the Louvain method nearly outperforms all other methods for the task of identifying the differential sub-network as a module. From Table 2, we observe that the 3 community detection techniques have nearly perfect Recall scores but usually have relatively low Precision values. This indicates that these methods correctly identify all the nodes forming the differential sub-network but also detect a large quantity of false-positives in the densest module, thereby reducing the Precision values. The Louvain and Infomap methods are extremely fast and interestingly the Louvain method has highest Precision (0.887) which is at least 10% higher than dGHD algorithm and 5% higher than Closed-Form approach while identifying the dense differential sub-network in a sparse network (*d*=0.15,*d* ^′^=0.3) as shown in Table 2.

We observe that among the community detection techniques the Louvain method is the most efficient and is highly competitive with the dGHD algorithm but cannot outperform the Closed-Form approach on denser networks and Power Law graphs.

### Case study in glioma

As a case study, we performed the differential sub-networks analysis of two gene regulatory networks re-constructed from the glioma dataset available on the TCGA. It is well known that the majority of gliomas are divided into two main macro-categories according to the mutation of the gene IDH1 [15, 17, 55]. Therefore, an important biological question, that motivated the development of the reported methodology, was to identify the sub-networks of of transcription factors (TFs) having a different regulatory program in these two major conditions. We re-constructed two gene regulatory networks belonging to two different glioma subtypes: IDH-mutant and IDH-wild-type as reported in the “Methods” Section.

In our final networks we have 457 TFs and 4,085 targets. We observe that these networks consist of 13,683 unique connections for IDH-mutant and 14,158 for IDH-wild-type between TF-TF and TF-target. Using these networks, we construct two unipartite topological graphs as described in the Methods section for the 457 TFs. We then perform the proposed differential sub-network analysis to identify the TFs which are part of differential sub-networks in these topological graphs.

**Fig. 5:**
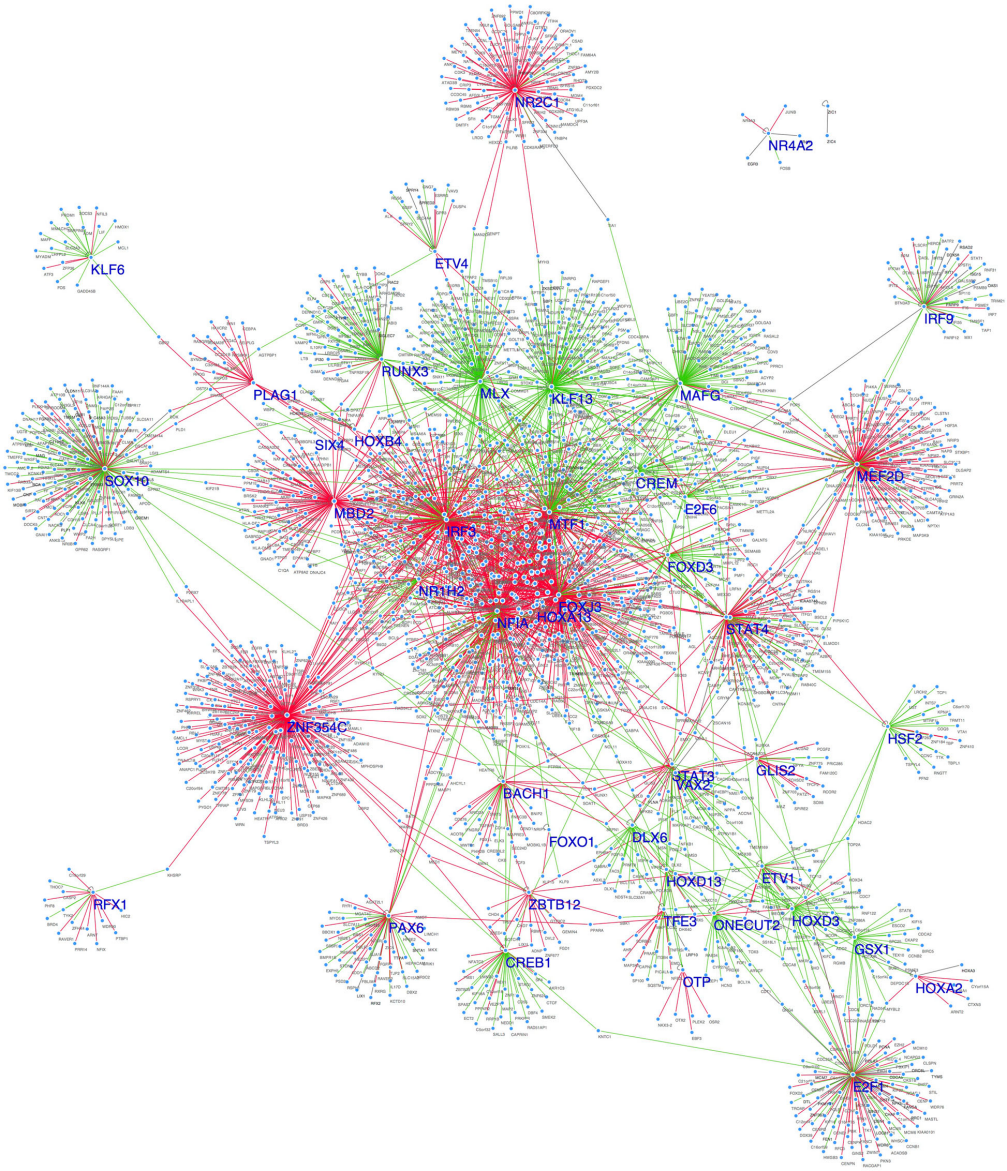
Differential sub-networks between IDH-mutant and IDH wild-type detected by the closed form approach. In *red* the connection present only in the IDH-mutant sub-network, while in *green* those present only in the IDH-wild-type sub-network. In *black* are represented common connections

Figure 5 shows the significant differential sub-networks and Table 3 reports the topmost TFs which are part of differential sub-networks as detected by our algorithm. In this table, GHD and *μ*_*π*_ represent the generalized Hamming Distance and its asymptotic mean between the subgraphs after removing the specific transcription factor in each row of the table. Additional file 1: Table S1 instead reports the results for all the 457 considered transcription factors.

**Table 3:** The top most different transcription factors detected between IDH-mutant and IDH-wildtype in the TCGA dataset

TF	Z-score	GHD	*μ*	MRA fdr
FOXD3	0.000	1.000	1.000	1.000E+00
FOXJ3	0.000	1.000	1.000	8.442E-03
MLX	0.000	1.000	1.000	8.075E-01
NFIA	0.000	1.000	1.000	4.502E-01
ETV1	0.062	0.058	0.058	1.000E+00
E2F1	0.085	0.058	0.058	1.007E-01
CREB1	0.208	0.058	0.058	8.580E-01
SOX10	0.234	0.058	0.058	8.442E-03
KLF13	0.338	1.000	0.278	1.240E-02
STAT3	0.354	0.058	0.058	8.442E-03
RUNX3	0.387	0.058	0.059	1.671E-02
IRF3	0.406	0.840	0.455	8.442E-03
ZNF354C	0.498	0.058	0.057	1.000E+00
HOXD13	0.540	0.059	0.059	2.492E-01
ZIC1	0.622	0.058	0.058	5.787E-02
HOXA2	0.700	0.059	0.059	1.405E-01
FOXO1	0.743	0.058	0.058	8.183E-02
MAFG	0.817	0.862	0.467	6.857E-01
RFX1	0.865	0.059	0.059	3.131E-01
NR1H2	0.871	0.058	0.058	8.176E-01
PAX6	1.003	0.058	0.057	4.147E-01
GLIS2	1.035	0.058	0.059	8.442E-03
NR4A2	1.118	0.058	0.058	1.000E+00
STAT4	1.137	0.848	0.486	9.615E-01
DLX6	1.208	0.058	0.059	1.000E+00
SIX4	1.232	0.058	0.058	1.000E+00
MEF2D	1.379	0.058	0.059	8.442E-03
MTF1	1.388	0.058	0.057	1.000E+00
MBD2	1.480	0.820	0.495	1.969E-01
OTP	1.493	0.058	0.057	2.970E-01
ETV4	1.529	0.059	0.059	2.122E-01
ZBTB12	1.566	0.194	0.189	4.255E-02
HOXB4	1.595	0.058	0.057	3.019E-01
PLAG1	1.622	0.195	0.190	3.434E-01
E2F6	1.668	0.197	0.192	8.442E-03
CREM	1.674	0.765	0.506	2.122E-01
IRF9	1.700	0.058	0.057	5.950E-02
KLF6	1.709	0.059	0.059	8.442E-03
TFE3	1.716	0.199	0.193	1.049E-01
HSF2	1.759	0.201	0.195	1.671E-02
NR2C1	1.800	0.058	0.058	2.122E-01
ONECUT2	1.804	0.202	0.196	3.657E-02
HOXD3	1.847	0.204	0.198	1.000E+00
BACH1	1.888	0.058	0.059	2.897E-01
GSX1	1.895	0.207	0.200	1.000E+00
HOXA13	1.930	0.058	0.057	1.000E+00
VAX2	1.937	0.208	0.201	1.609E-01

The columns reports the differential measures in terms of Z-score of the proposed differencing test (Eq. (2)), the GHD computed between the two networks, the mean of the null GHD distribution. The last column reports the False Discovery Rate of the GSEA enrichment obtained with a Master Regulator Analysis

In order to highlight the difference of Closed-Form approach with other standard network analysis methods, we also assembled a global glioma network using all the available transcriptional profiles using the same method described above and performed a master regulator analysis [38] with respect to the molecular phenotype under investigation, *i.e.* genes differentially expressed between IDH mutant and wild type. Master regulator analysis is extensively adopted to identify TFs that act as principal regulators in driving the phenotype from one condition to another.

Interestingly, among the topmost TFs (out of 457) forming the differential sub-networks, we found several genes known to have a central role in controlling specific glioma subtypes as well as novel candidates that deserve further biological validation. In particular, differential network analysis reveals that the sub-network of STAT3 is one the most different between IDH-mutant and IDH-wild-type networks and a particularly significant Master Regulator of this wild-type phenotype. Members of our group have previously shown that STAT3, together with C/EBP *β*, is a key regulator of the mesenchymal differentiation and predicts the poor clinical outcome of IDH-wild-type gliomas [38]. Another key regulator of the IDH-wild-type gliomas was recently reported by using an integrative functional copy number analysis is the set of HOXA genes [17]. Moreover, another key network hub that the algorithm detects as different is SOX10 which appears to be an active master regulator of the IDH-mutant phenotype. We recently reported that the GCIMP-low subgroup in the IDH-mutant cohort can mediated by loss of CpG methylation and binding of SOX factors [17]. Furthermore, our algorithm identifies methyl-CpG-binding domain protein 2 (MBD2) as a differential network hub. In particular, MBD2 has no links in the IDH-wild-type network whereas it is highly connected in the IDH-mutant network where it is characterized by the CpG island methylator phenotype (GCIMP) [56]. Further investigation is needed to claim such a hypothesis as MBD2 is known also as a mediator of the epigenetic gene regulation and its role in Glioblastoma is being studied as its over-expression may drive tumor growth by suppressing the anti-angiogenic activity of key tumor suppressors [57].

The differential network method highlights several other TFs as hubs of differential sub-networks which are not detected with standard MRA. For example, ETV1 and ETV4 which are over-expressed in gliomas of the Codel subtype carrying the mutation of the CIC gene [58]. Another differential sub-network hub not detected by standard MRA is the tumor suppressor RFX1 whch has been identified as an important target/regulator of the malignancy of Glioblastoma [59], where as the cell cycle regulators such as E2F1 and E2F1, which play a role in progression of IDH-mutant glioma are also detected by the Closed-Form algorithm [60].

An important warning that we want to mention is the presence of potential confounding effects due to the adopted dataset obtained by merging the expression profiles from two different platforms. With the additional difficulty that the distribution between IDH-wild-type tumors and IDH-mutated tumors is unequal between the two platforms (92% of microarray data are wild-type). We adopted this integrated dataset in order to build the two IDH networks and the global glioma network. The main computation in this case is the estimation of the mutual information between pairs of gene profiles (variables) in a set of observations (patients) and each individual pair of values is always extracted in the same platform. We used a robust k-nearest neighbor estimator proposed in [61] available in the PARMIGENE R package [62]. This estimator is not based on binning of values and is non parametric, working on the geometry of the scatterplot of each pair of gene expression values. Therefore, each observation (sample) can be seen as another evidence of dependency (or in-dependency) between the variables regardless to the platform. Although, we found this merged dataset useful for the estimation of dependencies between genes, its adoption for deriving conclusions in terms of sample groups and pathway analysis should be made with caution.

As a further independent experiment, we performed the same analysis using the REMBRANDT dataset with the network differential analysis on the two networks independently built with ARACNe and the Master Regulator Analysis on the global network. The Table 4 reports the results for the most different TF sub-networks detected by the Closed-Form algorithm on this dataset. Interestingly of the top nine differential nodes obtained in the TCGA dataset five (FOXJ3, NFIA, CREB1, SOX10, KLF13) are also detected as significant in the REMBRANDT dataset suggesting that these TFs have a very different regulatory program in glioma subtypes. Moreover, differently from the TCGA experiment, we observe a significant overlap between the results of Closed-Form and that of the MRA. In particular 70 of the 75 nodes forming the differential sub-networks are also enriched in the MRA (*p*-value of the Fisher exact test: 3.3810^−9^. However, in this case the number of significant master regulators is considerably higher than that obtained in the TCGA case (297 vs. 144).

**Table 4:** The top most different transcription factors detected between IDH-mutant and IDH-wildtype in the REMBRANDT dataset

TF	Z-score	GHD	*μ*	MRA fdr
MGA	0.000	1.000	1.000	2.166E-03
TEAD1	0.000	1.000	1.000	8.017E-04
FOS	0.000	1.000	1.000	5.137E-04
JUNB	0.000	1.000	1.000	5.137E-04
MEF2C	0.015	0.015	0.015	8.001E-04
LEF1	0.058	0.014	0.014	5.137E-04
NEUROD2	0.096	0.016	0.016	1.221E-03
EGR2	0.110	0.013	0.013	6.263E-03
JUN	0.123	0.333	0.500	5.137E-04
ARX	0.144	0.012	0.012	9.301E-02
BBX	0.173	0.012	0.012	7.333E-04
TCF3	0.198	0.011	0.011	5.137E-04
LHX6	0.205	0.017	0.017	8.492E-04
EGR1	0.211	0.011	0.011	9.696E-03
BCL6B	0.214	0.011	0.011	5.137E-04
E2F2	0.217	0.011	0.011	7.786E-04
E2F7	0.220	0.012	0.012	5.137E-04
E2F8	0.223	0.012	0.012	5.137E-04
ELF4	0.226	0.012	0.012	5.137E-04
ETV5	0.229	0.013	0.012	5.137E-04
FLI1	0.232	0.013	0.013	5.137E-04
FOXG1	0.235	0.013	0.013	1.000E+00
HOXD9	0.239	0.014	0.014	9.728E-04
ID4	0.242	0.014	0.014	7.786E-04
IRF8	0.246	0.014	0.014	2.393E-02
MYBL2	0.250	0.015	0.015	5.137E-04
NFIA	0.254	0.015	0.015	4.085E-03
NFIB	0.258	0.016	0.016	7.796E-04
KLF13	0.258	0.360	0.515	8.001E-04
OLIG2	0.262	0.016	0.016	7.893E-02
PROX1	0.266	0.017	0.017	1.020E-02
SOX2	0.270	0.017	0.017	2.995E-03
TEF	0.275	0.018	0.018	8.221E-04
ZBTB7A	0.280	0.019	0.018	7.700E-04
ZIC1	0.284	0.019	0.019	7.700E-01
SOX13	0.295	0.021	0.020	8.086E-04
TCF7L2	0.300	0.021	0.021	7.487E-04
BCL6	0.305	0.022	0.022	5.137E-04
MAF	0.317	0.024	0.024	5.137E-04
CEBPB	0.330	0.024	0.024	5.137E-04
CEBPD	0.337	0.025	0.025	5.137E-04
HLF	0.344	0.018	0.018	3.029E-03
ELK1	0.349	0.025	0.025	8.017E-04
FOXJ3	0.369	0.027	0.026	5.137E-04
MTF1	0.377	0.028	0.027	5.137E-04
TP53	0.388	0.028	0.028	5.137E-04
GABPA	0.407	0.030	0.029	5.137E-04
CDC5L	0.417	0.031	0.031	7.899E-04
RORA	0.422	0.329	0.467	7.796E-04
IRF9	0.426	0.031	0.031	3.062E-03
STAT1	0.437	0.033	0.032	5.137E-04
CREB1	0.456	0.035	0.034	5.137E-04
SOX10	0.462	0.036	0.035	8.250E-04
HOXD1	0.475	0.038	0.037	5.137E-04
SOX8	0.479	0.038	0.037	1.760E-03
HOXD11	0.480	0.047	0.046	2.975E-02
NR2F2	0.490	0.042	0.041	5.186E-04
DLX1	0.491	0.046	0.045	7.700E-04
TCF12	0.493	0.040	0.040	9.117E-04
THRB	0.495	0.051	0.050	9.850E-04
DLX2	0.496	0.045	0.044	8.492E-04
HOXD10	0.498	0.050	0.049	5.137E-04
ATF5	0.505	0.057	0.055	5.137E-04
STAT4	0.515	0.055	0.054	9.220E-04
TBR1	0.519	0.020	0.020	9.272E-04
MESP1	0.521	0.092	0.087	8.746E-04
POU3F2	0.523	0.063	0.061	5.137E-04
TFEC	0.530	0.082	0.079	5.137E-04
TCF4	0.533	0.071	0.069	7.487E-04
ETS2	0.543	0.176	0.163	9.728E-04
CREM	0.558	0.110	0.104	5.140E-04
TP63	0.561	0.105	0.099	9.220E-04
STAT6	0.563	0.091	0.087	5.137E-04
NPAS2	0.575	0.136	0.127	1.889E-01
GLI3	0.601	0.313	0.455	4.663E-02

The columns reports the differential measures in terms of Z-score of the proposed differencing test (Eq. (2)), the GHD computed between the two networks, the mean of the null GHD distribution. The last column reports the False Discovery Rate of the GSEA enrichment obtained with a Master Regulator Analysis

## Conclusion

The comparison of gene expression profiles across different phenotypes is enabling the discovery of novel biomarkers for prognosis or diagnosis. They hold the key to identify novel targets for therapeutical intervention. In this paper, we proposed an improvement to the state-of-the-art for comparing two labeled/unlabeled graphs that are representative of two conditions (e.g. the macro-categories according to the mutation of the gene IDH1 in our case study) and identifying statistically significant differences in their topology. We used the centralized GHD (cGHD) metric [28] to calculate the distance between the two labeled networks. We proposed a Closed-Form approach, an improvement to the dGHD algorithm, to detect localized topological differences between paired networks. The Closed-Form approach calculates the closed-form contribution of each node in the cGHD metric and efficiently removes nodes with the smaller contributions in the cGHD value. From our experiments on scale free random geometric networks, we discovered that the Closed-Form approach was 10-15x faster than dGHD from a computational complexity point of view. For differential sub-network analysis in very sparse paired graphs, both the Closed-Form and dGHD methods had good predictive performance. They reached mean AUC values of ≈0.935 and ≈0.926 respectively for 100 random runs of simulation experiments. However, for relatively denser networks, the Closed-Form approach outperformed dGHD. The proposed method achieved a mean AUC of ≈0.877 while the dGHD technique reached a mean AUC of ≈0.724. The Closed-Form approach also achieved much higher Precision, Recall and Kappa values in comparison to the dGHD method for relatively denser networks.

We applied our algorithm to detect the main differences between the networks of IDH-mutant and IDH-wild-type glioma tumors and show that it correctly selects sub-networks centered on important key regulators of these two different subtypes. The adopted dataset is the result of the merging of two different profiling platforms and, as reported in the Results section, its use for other purposes should be made with caution. We also report the results on the same data using standard Master Regulator Analysis on a global network, and show the overlap between the experiments. Indeed, it is known that MRA tends to have many false positives due to correlations between TF profiles which could eventually attenuated with synergy and shadow analysis. On the contrary, the Closed-Form algorithm for network differencing tends to be more conservative as also suggested by the fact that only the significantly different sub-networks are detected in both datasets.

## Additional file

Additional file 1
**Table S1.** Description of data: GHD and MRA Results for all the 457 considered transcription factors on the TCGA and Rembrandt datasets. (XLSX 62.7 kb)

## Abbreviations

AUC: Area under the curve
GHD: Generalized hamming distance
FP: False positive
FPR: False positive rate
GSEA: Gene set enrichment analysis
MAD: Mean absolute difference
MR: Master regulator
MRA: Master regulator analysis
QAP: Quadratic assignment procedure
RG: Random geometric
ROC: Receiver operating characteristic
TF: Transcription factor
TO: Topological overlap
TP: True positive
TPR: True positive rate
